# Adherence to non-pharmaceutical interventions following COVID-19 vaccination: a federated cohort study

**DOI:** 10.1038/s41746-024-01223-4

**Published:** 2024-09-10

**Authors:** Benjamin Rader, Neil K. R. Sehgal, Julie Michelman, Stefan Mellem, Marinanicole D. Schultheiss, Tom Hoddes, Jamie MacFarlane, Geoff Clark, Shawn O’Banion, Paul Eastham, Gaurav Tuli, James A. Taylor, John S. Brownstein

**Affiliations:** 1 Boston Children’s Hospital, Boston, MA USA; 2 Harvard Medical School, Boston, MA USA; 3 University of Pennsylvania, Philadelphia, PA USA; 4 Google, Mountain View, CA USA

**Keywords:** Computational science, Epidemiology, Ethics, Data acquisition

## Abstract

In pandemic mitigation, strategies such as social distancing and mask-wearing are vital to prevent disease resurgence. Yet, monitoring adherence is challenging, as individuals might be reluctant to share behavioral data with public health authorities. To address this challenge and demonstrate a framework for conducting observational research with sensitive data in a privacy-conscious manner, we employ a privacy-centric epidemiological study design: the federated cohort. This approach leverages recent computational advances to allow for distributed participants to contribute to a prospective, observational research study while maintaining full control of their data. We apply this strategy here to explore pandemic intervention adherence patterns. Participants (*n* = 3808) were enrolled in our federated cohort via the “Google Health Studies” mobile application. Participants completed weekly surveys and contributed empirically measured mobility data from their Android devices between November 2020 to August 2021. Using federated analytics, differential privacy, and secure aggregation, we analyzed data in five 6-week periods, encompassing the pre- and post-vaccination phases. Our results showed that participants largely utilized non-pharmaceutical intervention strategies until they were fully vaccinated against COVID-19, except for individuals without plans to become vaccinated. Furthermore, this project offers a blueprint for conducting a federated cohort study and engaging in privacy-preserving research during a public health emergency.

## Introduction

Individual-level uptake of non-pharmaceutical interventions (e.g., mask-wearing and social distancing) plays an important role in mitigating infectious disease transmission, but capturing and measuring levels of adherence to these behaviors can be difficult^1,2^. Throughout the COVID-19 pandemic, public health practitioners utilized population-level metrics as a proxy to measure individual NPI adherence^1,2^. Aggregate mobility data (e.g., Google Community Mobility and Safegraph Place Data), which synthesize signals from users’ mobile devices, improve our understanding of physical distancing and ability to generate models of disease dynamics^3^. However, in order to preserve the privacy of contributing individuals, meaningful mobility signals are generally only available in aggregate (e.g., at city, state, or country level), which obscures individual heterogeneity and makes it impossible to tie mobility changes to outcomes in the same individual^4^. Similar aggregate measures are often reported for vaccination levels, mask-wearing, and other important COVID-19-related behaviors^5,6^. Researchers have the option of utilizing self-report data when studying individual-level NPI adherence, but current self-report collection mechanisms (e.g., surveys) pose a threat to privacy which may result in discrepancies between reported and observed behavior, especially for sensitive questions^7^.

To overcome these obstacles, we employ a privacy-preserving scientific study framework that utilizes a mobile device application (app) to capture behavioral trends and NPI uptake with additional participant data protections. Through this lens, we utilize a federated cohort study—a privacy-centric approach to conducting clinical and observational research that allows distributed individuals to participate in a study while maintaining full control of their data. We deploy this study design to analyze COVID-19 disease and behaviors with the goal of demonstrating the feasibility and flexibility of conducting privacy-centric research during pandemic scenarios.

Leveraging recent advances in secure multi-party computation, we build an epidemiological cohort contributing data on sensitive COVID-19 metrics (e.g., individual mobility, masking, vaccination) without requiring research participants to grant the researchers or Google access to their raw, unaggregated data^8^. Our study was hosted in the “Google Health Studies” app—a platform that allows Android users to opt in and contribute to clinical research from their mobile devices. We performed aggregate analysis over application data using federated analytics (FA), a decentralized approach that enables the computation of epidemiological effect estimates across data from many participants’ devices while keeping individual-level data local, only contributing raw application data to aggregated results. Additionally, statistical noise was added to these aggregates to provide a rigorous differential privacy (DP) guarantee^9^.

Utilizing a privacy-preserving federated cohort, we measure NPI uptake before and after receiving the COVID-19 vaccine. In particular, there is concern that individuals may abandon adherence to NPIs following partial vaccination against COVID-19, which can lead to epidemic resurgence even in settings with high levels of vaccination^10^. Additionally, in the U.S., there is broad heterogeneity in understanding the necessity of COVID-19 mitigation measures after partial vaccination—a perspective that continues to be complicated by additional recommendations for booster doses^11^. Here, we use a combination of empirical and self-reported measures of NPI adherence to assess changes before and after COVID-19 vaccination.

## Results

### Overview

A prospective, observational, federated cohort study was conducted between November 2020 and August 2021 among US adults with access to an Android (operating system 7.0 or later) mobile device. Instead of pooling individual data into a central research database, the prospective federated cohort study empowered participants to remotely contribute to observational research while retaining full control of their data. Multiple privacy-focused technologies were employed to allow for the precise calculation of study endpoints from participant exposures and outcomes without requiring direct data-sharing.

### Participation

Of 9318 consented participants, 8049 completed the baseline survey, 5952 contributed to the initial FA queries, and 3808 contributed to the final FA query (Study Flow Diagram in Supplementary Fig. 1). All participant estimates reflect DP noise. A majority of participants were 18–39 years old, White, and male (Table 1). They represented almost all US states and the District of Columbia. In general, the characteristics of participants eligible to provide outcome data were similar to those of enrolled individuals.

**Table 1:** Characteristics of enrolled participants and those eligible to respond to demographic queries

Characteristic	Eligible to respond to baseline characteristics queries^a^	Eligible to respond to outcome queries^a^
Maximum recorded *N*^b^	5952	3808
Age, number of valid responses^c^	5440	3676
18–29 years	32.6%	28.8%
30–39 years	32.4%	35.4%
40–49 years	19.6%	19.5%
50–59 years	9.0%	9.9%
60–69 years	4.3%	4.4%
70–79 years	1.6%	2.0%
80+ years	0.4%	0.1%
State of residence, number of valid responses	5584	3792
Race/ethnicity, number of valid responses	5952	3808
American Indian/Alaska Native	2.2%	0.8%
Asian	8.1%	7.6%
Black/African American	4.8%	4.2%
Hispanic/Latino ethnicity	8.6%	6.7%
Pacific Islander/ Native Hawaiian	0.5%	0.8%
White	75.8%	79.8%
Gender, number of valid responses	5,504	3,724
Male	71.1%	72.5%
Female	26.7%	25.3%
Non-binary	2.1%	2.1%

^a^Eligible to respond indicates that a participant had the app installed on their mobile device and the device responded to any query during the time when baseline or outcome data were being collected.

^b^FA aggregation treats every query as independent and does not collect unique participant identifiers. Maximum recorded *N* therefore reflects the *N* from the individual query with the most participants, not total number of unique contributors, which cannot be computed across multiple independent queries (see privacy appendix for more detail).

^c^Valid responses are the number of responses to a particular query (e.g., age query). The totals do not include “missing” or invalid responses.

### Outcome metrics collection via a federated cohort study

Data on main study outcomes: time at work and home, use of mask-wearing and social distancing, and places visited were grouped by time period and classified by vaccination status. The number of participants who provided data in at least 1 week for a given 6-week time period varied slightly by outcome. In addition, since enrollment and participation were rolling, those who enrolled later did not contribute outcome data during earlier time periods, and vice-versa. The average number of participants who contributed outcome data during each of the five time periods was 1907 for time period 1; 2130 for time period 2; 2332 for time period 3; 2470 for time period 4; and 1807 for time period 5. During time period 4, when >90% of participants were actively enrolled, outcome data were collected on 30.4% of those enrolled.

### Vaccination and non-pharmaceutical intervention uptake over time and by vaccination status

**Fig. 1:**
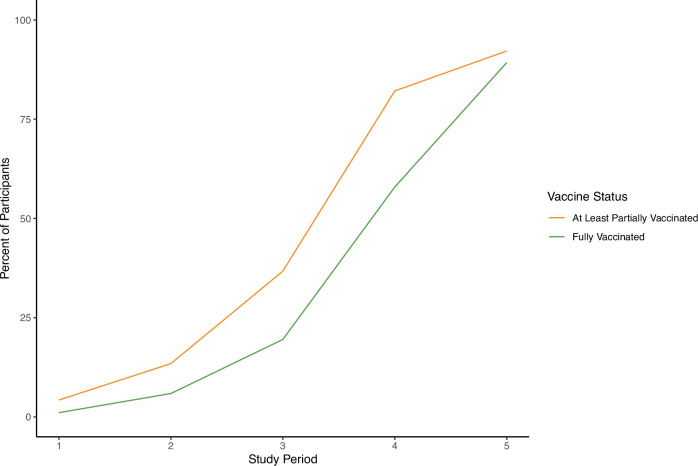
Percent of participants who were at least partially vaccinated or fully vaccinated by study time period. Vaccination status of study participants over five time periods between December 9, 2020 and July 6, 2021.

**Fig. 2:**
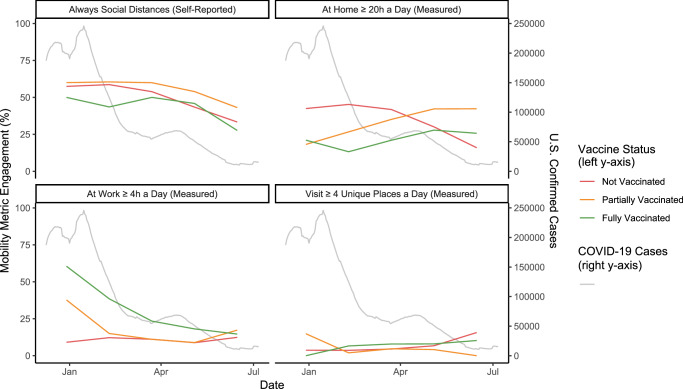
Participant engagement in mobility-driven non-pharmaceutical COVID-19 interventions. Four panels represent the percentage of participants who participated in various metrics of self-reported and empirical measurements of mobility over five time periods between December 9, 2020 and July 6, 2021.

Corresponding to the roll-out of COVID-19 vaccines in the US, the percentage of participants who were at least partially vaccinated or fully vaccinated increased dramatically during the study period (Fig. 1). The use of specific NPIs that modified the risk of developing COVID-19 by vaccination status during each time period are shown in Fig. 2.

Partially vaccinated participants tended to report always wearing a mask and practicing social distancing more than those who were fully vaccinated, and at similar rates or more than unvaccinated participants. For the mobility measures, including daily unique places visited, daily time at work, and daily time at home, relationships between participants in different vaccination status groups were more nuanced. In time period 1, 9.1% of unvaccinated participants were at work ≥4 h/day compared to 37.3% of those partially vaccinated (*χ*^2^, *P* < 0.001), but there were no other significant differences between these groups for any other time period. Those who were fully vaccinated had significantly higher rates of being at work ≥4 h/day than participants who were unvaccinated, or those who were partially vaccinated for all time periods except time period 5.

Additionally, 14.9% of partially vaccinated participants visited ≥4 places/day in time period 1, compared to 3.7% (*χ*^2^, *P* < 0.001) for those who were unvaccinated, and 0% (*χ*^2^, *P* = 0.199) of those who were fully vaccinated. However, the percentage of partially vaccinated participants visiting ≥4 places/day dropped and was comparable or significantly less than those who were unvaccinated or fully vaccinated for time periods 2–5.

For every behavior assessed, partially vaccinated participants were significantly more likely to adopt NPI behaviors than fully vaccinated participants. During time periods 4 and 5, future vaccination intentions were obtained from participants who were unvaccinated. There were an average number of 912 and 288 responses, respectively, for queries related to the use of NPIs. During time period 4 (April 14–May 25, 2021), 44.0% of unvaccinated participants planned to receive the vaccine when it was available to them, and 56.0% had no plans to vaccinate. By time period 5 (May 26–July 6, 2021), only 23.1% of those unvaccinated planned to receive the COVID-19 vaccine, while 76.9% had no vaccination plans. Those with plans to vaccinate reported higher mask-wearing, social distancing, and time spent at home, than those who did not (Table 2).

**Table 2:** Overall comparison of behaviors thought to modify the risk of COVID-19 by vaccination status

Behavior	Partially vaccinated vs. unvaccinated participants^a^	Partially vaccinated vs. unvaccinated participants (limited to those planning to get vaccinated when available) ^a^	Partially vaccinated vs. fully vaccinated participants^a^	Plans to vaccinate vs. no plans to vaccinate among unvaccinated participants^a^
Visiting <4 places/day	1.15 (0.82, 1.64)	1.92 (0.93, 3.57)	1.61 (1.22, 2.17)	1.43 (0.48, 4.00)
Reported “always” wearing face mask in public	1.53 (1.36, 1.72)	0.82 (0.64, 1.05)	1.69 (1.52, 1.89)	3.72 (2.66, 5.36)
Reported “always” practicing social distancing	1.26 (1.16, 1.37)	0.95 (0.79, 1.14)	1.47 (1.33, 1.59)	2.45 (1.77, 3.41)
At work <4h/day	0.69 (0.58, 0.83)	0.37 (0.18, 0.80)	2.08 (1.75, 2.50)	0.75 (0.42, 1.35)
At home ≥20 h/day	0.83 (0.76, 0.91)	1.23 (1.0, 1.5)	1.61 (1.45, 1.79)	2.62 (1.60, 4.41)

^a^Values presented are odds ratios (95% confidence interval) adjusted for the time period.

### Non-pharmaceutical intervention uptake and vaccination adjusting for the time period

Logistic regression results adjusting for time period suggest that compared to the unvaccinated, partially vaccinated participants were significantly more likely to report NPI behaviors and going to work (Table 2). However, when limited to the unvaccinated group to just those who plan to receive the vaccine, we find no statistically significant difference between the two groups.

## Discussion

In this study, we employ a federated cohort study framework—a privacy-centric methodology for conducting epidemiological research. By integrating federated analytics, secure aggregation, and differential privacy, we were able to gather mobility signals and responses from behavioral questionnaires while ensuring participant control of their own sensitive data. This approach strikes a balance between ensuring participant privacy and providing a generalizable framework for analyzing adherence to disease mitigation strategies during public health emergencies.

Using empirical measurements and self-reports between December 2020 and July 2021, we show that the use of NPIs was largely consistent with public health recommendations, with high uptake among those planning to vaccinate and relaxation once fully vaccinated. Unvaccinated individuals who had no plans to vaccinate were a notable exception to this trend and displayed preventive behavior patterns similar to vaccinated individuals. Towards the end of the study period, there is some evidence that partially vaccinated participants had increased time spent at work, but visited fewer unique places than those who are fully vaccinated. This may suggest some participants were compelled to return to work or engage in other essential activities following partial vaccination but otherwise maintained social distancing vigilance.

Over time, there have been observations of decreased adherence to COVID-19-recommended mitigation strategies such as masking and social distancing^12^. Two prominent hypotheses have been proposed to explain this trend: “pandemic fatigue” and “risk compensation”^12,13^. The latter describes a process by which people adjust competing behaviors to minimize risk, and may manifest as a reduction in adherence to risk-reducing NPIs following COVID-19 vaccination^11^. Here we find evidence supporting both hypotheses. Over the five study periods, we observed a reduction of self-reported adherence to social distancing and mask-wearing among fully vaccinated, partially vaccinated, and non-vaccinated participants. This reduction coincided with large decreases in COVID-19 cases in the U.S. However, we also found a large effect of NPI reduction when individuals moved from partially to fully vaccinated, supporting the idea of risk compensation. Interestingly, in early study periods, partially vaccinated participants appear more similar to non-vaccinated strata (which contain many yet-to-be-vaccinated individuals), suggesting in the context of COVID-19 vaccines, risk compensation is a binary (fully vs. unvaccinated) non-linear process and many who intended to get vaccinated only felt protected enough to change behavior after a full vaccine course. It will be important to monitor these changes as periodic booster recommendations modify what it means to be up-to-date with COVID-19 vaccination.

There are several limitations associated with this study. Our study was of a convenience sample, and participants are not representative of the public as a whole (e.g., ~71% of enrollees were male); therefore, our findings may not be broadly generalizable. The privacy-preserving methodologies used also introduced noise and bias. We collected only pre-planned aggregate responses (FA prevents observation of individuals’ data), and therefore, additional control of confounding could not be conducted. Additionally, DP introduces noise to each cell independent of the number of cells, so as stratifying variables are added, per-cell counts and the resulting signal-to-noise ratio decrease. FA also introduces the risk of bias from uneven participation of enrolled devices in the FA process, owing to different device capabilities and availability^14^. Third, we had a ~30% outcome data-capture rate arising from a combination of missing records (e.g., due to survey nonresponse, turned-off devices, and/or missing app permissions) and device availability. As data were stored securely on participant’s devices and not in a centralized repository, data were lost for individuals who uninstalled the Google Health Studies app between the end of their 6-month study participation and execution of our post-study FA aggregation. Fourth, our federated survey aimed to overcome response bias from self-reports by creating a private environment that facilitates candor in responses. While it is possible our responses were still affected by social desirability bias to some degree, we found general trends between self-reported mobility metrics (e.g., social distancing) and measured ones (e.g., time spent at work) to be consistent. However, our empirical measures of mobility were only for participants’ powered-on devices, and not participants themselves.

Surveys and participatory surveillance systems have the potential to fill large data gaps left by traditional infectious disease surveillance infrastructure^15^. However, privacy concerns continue to grow as both conventional and non-conventional data collection systems incorporate potentially invasive data streams (e.g., mobility) in their models^16^. In response, we employed an observational research framework, the federated cohort study. Through this method, we were able to safeguard participants’ data, minimize the social desirability bias often tied to self-reports, and directly measure non-pharmaceutical interventions. We found self-reported and empirical evidence that during the early stage of the pandemic, except for the unvaccinated who did not plan to become vaccinated, individuals largely followed public health guidelines and utilized NPIs. The success of our approach suggests that privacy-preserving technologies have the potential to be incorporated into epidemiological studies and pandemic surveillance systems, providing accurate results without compromising individual data.

## Methods

### Enrollment

All study activities, including enrollment and data collection, were conducted on the “Google Health Studies” mobile app, downloaded from the Google Play Store. A convenience sample was recruited via blog posts, webinars, and online advertisements. Those interested were instructed to download the app and enroll in the “Respiratory Health Study”, where a study description was provided. Eligibility was confirmed by reporting state of residence and age (minimum age of 18–21 years varied with each state’s age of majority). If age and state requirements were met, participants were provided with an in-app consent form and phone number to contact with additional questions. Consent was indicated by clicking a box at the form bottom. Participants were enrolled for a 6-month period.

The study (Clinicaltrials.gov: NCT04663776) was approved by the Boston Children’s Hospital institutional review board (IRB-P00036213), with a waiver for documentation of written informed consent.

### Measures

Following consent, participants completed an initial survey with demographics (e.g., gender and race/ethnicity) and their work and home addresses. Individuals who did not complete the initial survey and/or did not grant mobility data permissions were excluded.

Each week, while enrolled, participants were sent an app notification to complete a survey and were asked about the use of preventive measures, including consciously practicing social distancing and wearing face masks while in public. Participants responded using a 5-point Likert scale, ranging from “never” to “always.” Beginning in March 2021, participants were asked whether they received a COVID-19 vaccine and, if so, the date of the first dose, second dose (if received) or indicate a second dose was not required based on the specific vaccine received. Unvaccinated participants were asked if they planned to receive the vaccine when available.

Mobility data were recorded daily from device sensors (e.g., GPS)^17^ on the following measures: time spent at home, time spent at work (both based on the participant’s reported work and home addresses), and number of unique places visited. Each completed survey was associated with average daily mobility measurements from the 14-day period prior to the survey completion date. Data were discarded if less than 7 days were measured in a 14-day period.

### Privacy overview

**Fig. 3:**
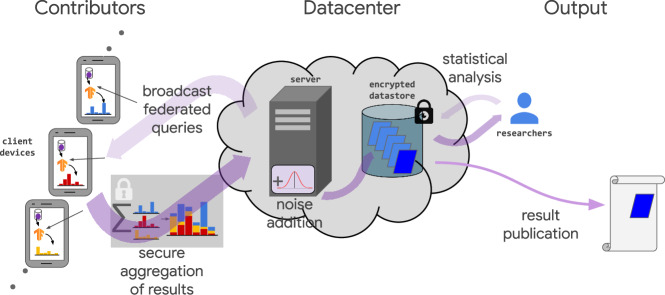
Federated Cohort Study Framework. *Federated analytics* (FA) refers to the process of broadcasting statistical computations (“federated queries”) to client devices, executing those computations locally over each device’s raw data, and aggregating these local results without ever making any data from individual devices available to engineers or researchers. In this study, the results are securely aggregated using a cryptographic protocol (SecAgg) that prevents the central server from learning any individual device’s results^18,19^. On the server, noise is drawn from a Laplace distribution and added to the aggregates to achieve differential privacy^23^ before writing them into an encrypted datastore. Only these differentially private aggregates are accessible to researchers for further analysis and publication.

The Google Health Studies app employs three primary technologies to protect participant privacy (Fig. 3) and control of raw data: federated analytics (FA), secure aggregation (SecAgg), and differential privacy (DP). Detailed descriptions of privacy technologies employed are available in “Privacy details” section.

FA is a method for analyzing raw data stored locally on participants’ devices without any centralized server or researcher accessing that raw data. Instead, local computations are conducted on individual devices, and statistics like counts and quantiles are aggregated from them without revealing any individual-level values to engineers or researchers^18^. Via the SecAgg protocol, individual-level values are not revealed to any server, even transiently. Our implementation of the SecAgg protocol is secure against an honest but curious adversary with a joint view of the server and up to 20% of client devices contributing data to the aggregation; i.e., a “passive” adversary who possesses such a view but does not actively interfere with the protocol cannot gain any additional information about the other contributors’ inputs. It is also robust against up to 1/3 of the clients dropping out during the protocol execution and maintains input privacy regardless of the number of clients that drop out^18–20^.

In addition to collecting aggregate results via SecAgg, we applied differential privacy (DP) to the aggregate output as a secondary mechanism to promote privacy^21,22^. Note that informed consent communications did not discuss DP, and participants’ opt-in was not contingent on DP guarantees. DP is a rigorous, mathematical definition of privacy that characterizes the impact a single participant’s contribution can have on the result of the computation^23^. DP data-processing mechanisms are randomizing; they guarantee statistically similar aggregate outputs regardless of whether a single participant’s data was included, thereby ensuring no individual’s contribution can be inferred with certainty from any single output. To achieve this, random noise is drawn from a Laplace distribution and added to the aggregate data before making it visible to researchers.

Intuitively, the injection of noise to achieve DP adds a degree of uncertainty to the collected data that is calibrated to give each individual participant *plausible deniability* about their own contribution while still preserving scientific utility of the aggregate result. This privacy-accuracy tradeoff is illustrated in Supplementary Fig. 2.

### Privacy details

Our FA framework imposed some restrictions on the study:

1. Participant data is only accessible to researchers via aggregation operations—summation in particular. We, therefore, estimate continuous variables, such as time spent at home or unique places visited, by bucketing these data and aggregating histograms across participants.
2. To further protect participant privacy, our FA infrastructure intentionally breaks any connection between participant identifiers and the aggregate results to which they contribute. While each participant can contribute at most once to each FA query, the lack of individual identifiers prevents us from computing the number of unique contributors across those multiple queries.
3. Data recording at participants’ devices was decoupled from aggregate data collection at the server, so participants who filled out surveys or activity data did not actually contribute data to the study results if they deleted the application or that data from their devices before FA aggregations could be performed.
4. Only participants whose mobile devices were reachable and could rendezvous with other participants during securely aggregated FA aggregations (arranged in repeated minutes-long windows over the course of a few weeks) could contribute their data to the analysis. To minimize the impact on participants’ mobile devices, devices were only eligible to contribute when idle, charging, and connected to an unmetered network.

In practice, for contemporaneous FA queries A and B, the symmetric difference between participating populations (i.e., the number of participants who contribute to only query A or only to query B) should be small because the technical eligibility criteria enumerated in the final bullet above are shared between queries, and the availability of data for contemporaneous queries (e.g., whether the contributor has filled out surveys during the queried time period) are generally correlated, so any device able to contribute to query A can likely contribute to query B. Since our final analysis queries for this study were run contemporaneously, we expect their contributors to be mostly overlapping, mitigating limitation #2.

Alongside data-recording nonresponse (that is, not completing surveys, declining to grant location permissions to the study application, leaving the mobile device turned off, etc.), points #3 and #4 contributed significantly to the study’s ~30% data-capture rate. In addition, point #4 introduces an unquantified risk of bias from uneven participation by devices with different availability. For future federated studies, these two issues could be mitigated via both technical and operational changes, including:

- improvements to the SecAgg protocol, such as SubGraph SecAgg^24^ that would reduce the cost of coordination among contributing devices
- alterations to the device eligibility criteria to allow more devices to attempt aggregation more frequently
- aggregation of data throughout the study rather than after its completion, reducing the chance of data loss due to app uninstallation

Differential privacy provides participants plausible deniability about the data that they contributed to a computation by offering a rigorous, mathematical upper bound on the impact that a single participant’s contribution can have on the result of the computation^23^. A DP mechanism M is considered to be ε-DP if, for all pairs of adjacent datasets D and D’ that differ only by changing, adding, or removing one participant’s data and for all possible outputs S, Pr[M(D) = S] ≤ e^ε^⋅Pr[M(D′) = S]^23^.

Intuitively, *ε* defines a ratio that bounds how much a single contributor’s data can affect the probability of any given result. For example, if *ε* = ln(3), no change to a single participant’s contribution can change the probability of any result by more than 3×. For a binary question, this is the level of protection provided by a randomized response in which the participant flips a coin and answers truthfully if heads and randomly if tails, since a participant whose true answer is Yes has a probability of 75% of answer Yes and 25% for answering No, and vice versa if their true answer is No.

For each FA aggregation, we apply the Laplace mechanism^2^ to the server-side aggregates to guarantee differential privacy of the users who contributed to that FA aggregation. The Laplace mechanism involves addition of noise drawn from the Laplace distribution and scaled according to the sensitivity of the output to an individual participant’s contributions and the desired privacy parameter *ε*. We add this noise to the server-side, securely aggregated data before making it visible to researchers.

Critically, the addition of noise to the aggregated results limited researchers’ ability to slice aggregates to control for arbitrary confounders, since more slices with the same privacy parameter ε implies a lower signal-to-noise ratio, eventually washing out the data’s utility. We, therefore, limit our aggregates to bivariate comparisons.

Since noise-addition imposes a tradeoff between utility and privacy guarantees, we, therefore, lean toward utility in our strategy selection for differential privacy. We choose a strong *ε* value of ln(3) to protect each participant’s contribution to each bivariate aggregation but acknowledge that each participant can contribute to multiple bivariate comparisons and that these comparisons have some correlation.

### Analysis

The analysis focused on the association between COVID-19 vaccination status and NPIs, including self-reported use of face masks, conscious social distancing, and mobility measures. Primary study outcomes were: daily time at work (<4 h vs. ≥4 h), daily time at home (≥20 h vs. <20 h), daily number of unique places visited (<4 places vs. ≥4 places), self-reported use of face masks (“always” vs. less), and self-reported social distancing (“always” vs. less).

The primary study exposure was vaccination status. Participants were defined as unvaccinated until 11 days after an initial dose of COVID-19 vaccine, partially vaccinated from 11 days after dose 1 until 11 days after dose 2, and fully vaccinated when they were ≥11 days post vaccine dose 2. Individuals who indicated they received a single-dose version of the COVID-19 vaccine were classified as fully vaccinated ≥11 days after the dose.

During the study period, there were multiple changes in public policy, public health recommendations, and vaccine availability. To account for these changes, study data were grouped into 6-week time periods, beginning on December 9, 2020, paralleling the release of the COVID-19 vaccine in the US, through July 6, 2021. During each 6-week period, participants could contribute data on 0-6 weekly surveys. Due to FA, it was not possible to attribute multiple weekly surveys to a single participant. For each time period, the total number of responses for each dichotomously measured NPI was determined for each of the 3 possible vaccination statuses. We adjusted for repeated responses by dividing the total number of responses for a given exposure variable by the total number of participants who responded to at least one data query on that exposure variable during the time period. We divided the responses for each NPI- vaccination status pair by this correction factor and rounded to the nearest integer to approximate per-participant values.

Chi-square tests were used to assess differences in behaviors related to vaccination status during each time period. Separately, logistic regression models were used to identify overall differences in behaviors by vaccination status after adjusting for time period. Finally, logistic regression models were used to compare behaviors among the sub-groups of unvaccinated participants who did and did not plan to get vaccinated after adjusting for the time period. To account for idiosyncrasies introduced due to our privacy-preserving methodology, we used a non-parametric bootstrap with 10,000 iterations to construct 95% confidence intervals (Supplementary Note 1). Our use of federated analytics constrained our ability to adjust for multiple variables without compromising data utility, as adding confounders would have necessitated more data stratifications, diluting the signal under constant noise levels enforced by DP. Therefore, we limited our analyses to select key variables aggregated results across the time periods.

## Supplementary information

Supplemental Material

## Data Availability

Privacy analysis and participant consent did not account for the disclosure of aggregate data, and therefore, it is unavailable.

## Code availability

Code for specific implementations of data retrieval from the Google Health Studies app relies on internal Google libraries and is unavailable for sharing. Code and public data examples for implementing federated techniques in conjunction with differential privacy are available and described in ref. ^8^.
