# Supplementary material for: Adherence to non-pharmaceutical interventions following COVID-19 vaccination: a federated cohort study
Source: NPJ Digit Med. 2024 Sep 10;7:241. doi: 10.1038/s41746-024-01223-4 (PMC11384771; doi:10.1038/s41746-024-01223-4)
Supplement: Supplementary file 1 — Supplemental Material [file 41746_2024_1223_MOESM1_ESM.pdf]

Supplementary Note 1

To account for idiosyncrasies introduced due to our privacy-preserving methodology, we used a non-parametric bootstrap with 10,000 iterations to construct 95% confidence intervals through the percentile method. Our non-parametric bootstrap aimed to replicate what happened to the original data as closely as possible in an attempt to incorporate all known sources of noise: the statistical noise, differential privacy noise, repeated-response adjustment noise, and rounding steps. For a single bootstrap iteration, within each time period we draw a sample with replacement. We then added noise from a Laplace distribution calibrated for $\varepsilon = \ln(3)$ as described above, adjusted for repeated responses, and rounded before fitting a logistic regression.

Supplementary Figure 1. Participant Enrollment Flow

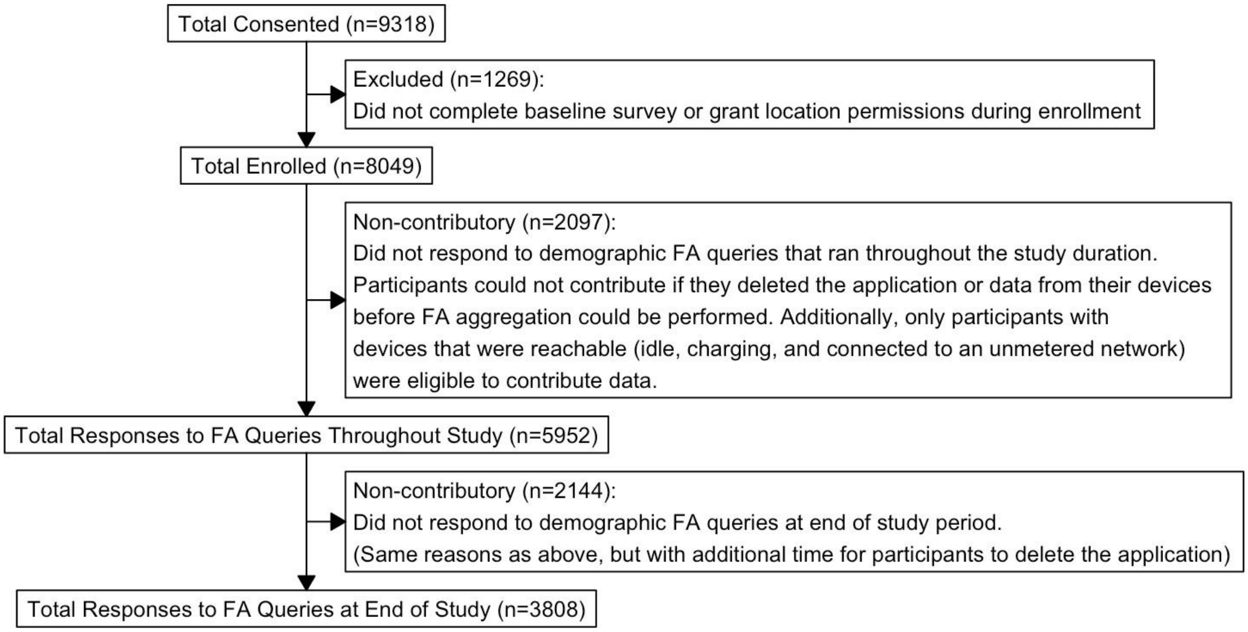

Enrollment, participation, and data query rates of study participants in the federated cohort study from November 2020 to August 2021. Of the 9,318 participants initially consented, 3,808 contributed to the final analysis. Note, all participant numbers are estimates that reflect differential privacy noise.

Supplementary Figure 2. Mean Absolute Error Variation by Epsilon

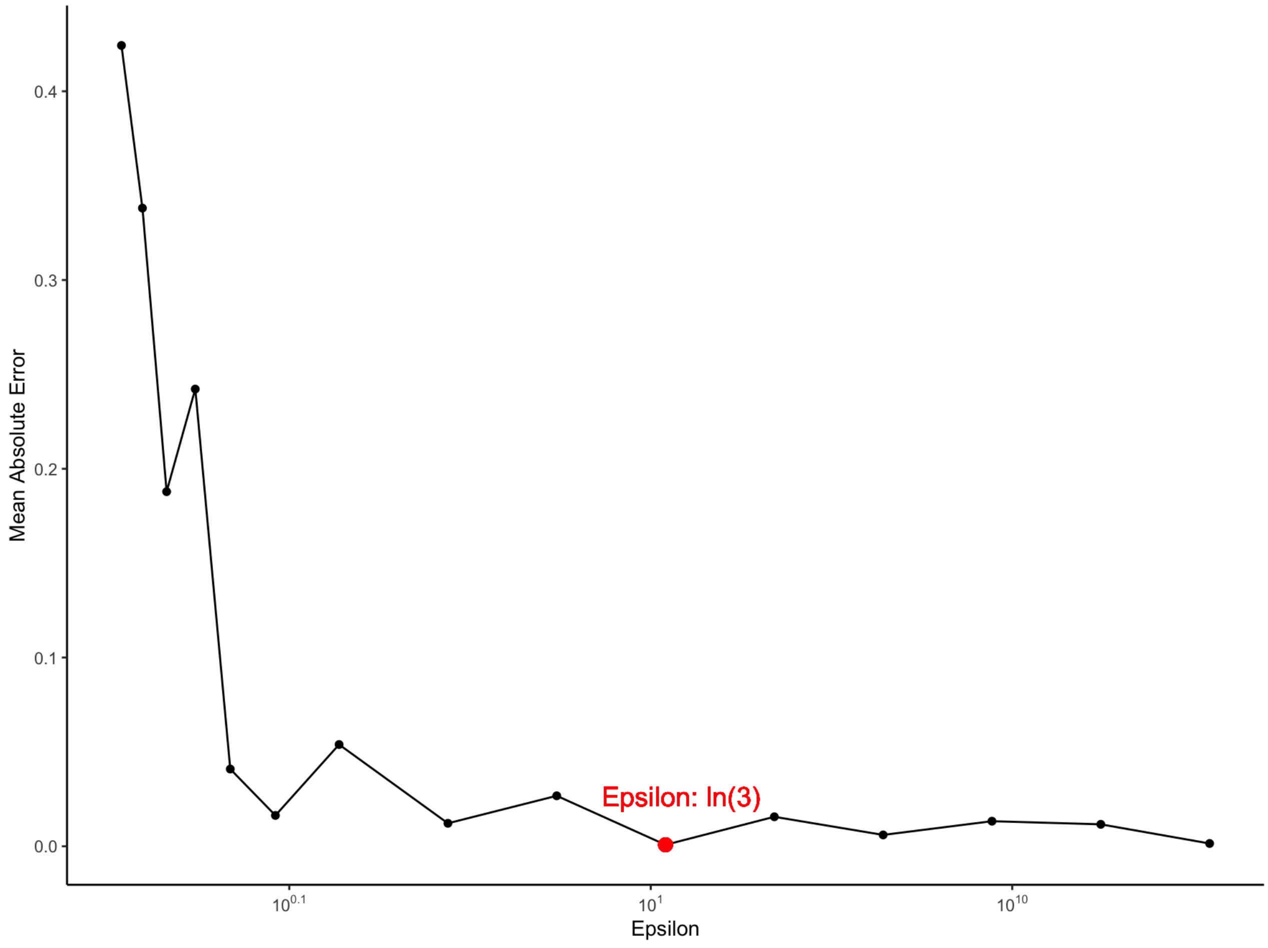

We examined the relationship between epsilon (ε) and error by modifying the value of epsilon in our non-parametric bootstrap. For a single bootstrap iteration, within each time period we drew a sample with replacement. We then added noise from a Laplace distribution, adjusted for repeated responses, and rounded before fitting a logistic regression. In our study, we chose a strong ε value of ln(3) to protect each participant's contribution to each bivariate aggregation.
